# Resin infiltration of deproteinised natural occlusal subsurface lesions improves initial quality of fissure sealing

**DOI:** 10.1038/ijos.2017.15

**Published:** 2017-06-16

**Authors:** Andrej M Kielbassa, Ina Ulrich, Rita Schmidl, Christoph Schüller, Wilhelm Frank, Vanessa D Werth

**Affiliations:** 1 Centre for Operative Dentistry, Periodontology, and Endodontology, University of Dental Medicine and Oral Health, Danube Private University (DPU), Krems, Austria; 2 Department of Applied Genetics and Cell Biology, UFT-Campus Tulln, University of Natural Resources and Life Sciences (BOKU), Vienna, Austria; 3 Centre for Preclinical Education, Department of Biostatistics, University of Dental Medicine and Oral Health, Danube Private University (DPU), Krems, Austria

**Keywords:** aprismatic enamel, fissure sealing, occlusal caries, resin infiltration, sodium hypochlorite

## Abstract

The aim of this *ex vivo* study was to evaluate the infiltration capability and rate of microleakage of a low-viscous resin infiltrant combined with a flowable composite resin (RI/CR) when used with deproteinised and etched occlusal subsurface lesions (International Caries Detection and Assessment System code 2). This combined treatment procedure was compared with the exclusive use of flowable composite resin (CR) for fissure sealing. Twenty premolars and 20 molars revealing non-cavitated occlusal carious lesions were randomly divided into two groups and were meticulously cleaned and deproteinised using NaOCl (2%). After etching with HCl (15%), 10 premolar and 10 molar lesions were infiltrated (Icon/DMG; rhodamine B isothiocyanate (RITC)-labelled) followed by fissure sealing (G-ænial Flo/GC; experimental group, RI/CR). In the control group (CR), the carious fissures were only sealed. Specimens were cut perpendicular to the occlusal surface and through the area of the highest demineralisation (DIAGNOdent pen, KaVo). Using confocal laser-scanning microscopy, the specimens were assessed with regard to the percentage of caries infiltration, marginal adaption and internal integrity. Within the CR group, the carious lesions were not infiltrated. Both premolar (57.9%±23.1%) and molar lesions (35.3%±22.1%) of the RI/CR group were uniformly infiltrated to a substantial extent, albeit with significant differences (*P*=0.034). Moreover, microleakage (*n*=1) and the occurrence of voids (*n*=2) were reduced in the RI/CR group compared with the CR group (5 and 17 specimens, respectively). The RI/CR approach increases the initial quality of fissure sealing and is recommended for the clinical control of occlusal caries.

## INTRODUCTION

It is generally accepted that the occlusal surfaces of premolars and molars are caries-prone and account for a majority of all carious lesions. Adhesive sealing of fissures using (un-)filled composite resins (CRs) has proven to be a preventive strategy with high efficacy^1^ and highly significant caries reduction rates.^2^ In specimens sealed clinically, bacteria located in the fissures were entrapped or explicitly reduced^3^ by the sealing procedure, thus indicating that any microbial activities will be stopped.

Moreover, with their pioneering clinical studies, Handelman *et al.*^4^ revealed as early as 1976 that sealing of incipient lesions decreased the number of cultivable microorganisms to a scattering fraction, and these authors did not observe progression of the sealed carious lesions radiographically nor clinically. This outcome was corroborated,^5^ and two long-term (10 year) clinical trials repeatedly reinforced these results. The majority of initially sound molars remained free of carious lesions,^6^ and even frank Class I cavities bonded and sealed with CR restorations (without removing any of the diseased dentine) were arrested.^7^ In particular, non-cavitated occlusal carious lesions may be treated by fissure sealing,^2, 8, 9^ thus using a non-drilling approach and replacing invasive procedures without any adverse consequences.^10^ Therefore, rather than waiting for any breakdown of occlusal surfaces (necessitating restorative care), an occlusal sealant is favourable to prevent lesion progress.

However, fissure morphology, material-related factors (that is, viscosity of the sealant), and procedural issues leading to voids (that is, entrapped air) contribute to reduced penetration depths,^11, 12, 13^ thus curtailing retention periods. Moreover, the longevity of the sealant restoration might be impeded in cases of incompletely occluded fissure systems. The same hypothesis applies to sealing of carious areas (demineralised and weakened enamel, softened dentine). Here incomplete sealing might fail under functional loading, possibly leading to “ice-cracking” of the restoration.^14^

To overcome these limitations, a complete filling down to the bottom of the fissure system and a thorough replenishment of the increased porosities within the subsurface crystalline structures of incipient carious lesions would seem favourable. A low-viscous resin infiltrant (RI) has been developed and penetrates and occludes the porous volume of subsurface lesions,^15, 16^ thus significantly increasing the microhardness of demineralised enamel (and restoring hardness of sound enamel).^17, 18^ Some preliminary investigations focussing on aspects of occlusal infiltration have presented promising outcomes both *in vitro*^19, 20^ and clinically.^21^ However, organic material (for example, proteins,^22, 23^ lipids,^22, 24^ and bacterial compounds^25^) often block the pores of natural subsurface lesions.^22^ Consequently, thorough cleaning of these lesions would seem imperative. Deproteinisation by means of sodium hypochlorite enhances remineralisation^26^ and accordingly increases infiltration depths after the use of even viscous resin materials.^27^

Against this background, the present study’s objective was to evaluate penetration and sealing efficacy of a low-viscous RI on deproteinised natural occlusal carious lesions of premolars and molars and to compare the respective outcome with the sealing effects of a flowable CR. Additionally, both marginal adaption and internal integrity (occurrence of voids) were evaluated in both groups using confocal laser-scanning microscopy (CLSM). We hypothesised (H_0_) that the RI/CR combination was comparable to the exclusive and conventional use of CR, and H_0_ was tested against the alternative hypothesis (H_A_) of a difference.

## MATERIALS AND METHODS

### Selection of teeth

A total of 40 extracted permanent human teeth (20 premolars and 20 molars) revealing non-cavitated white/brownish occlusal lesions (fissure caries) were used for the present *ex vivo* study. As parts of the human body no longer related to the individuals they were removed from, the respective teeth were applicable for medical research, and no ethical approval was necessary for the current investigation.^28^ The teeth were independently classified according to International Caries Detection and Assessment System (ICDAS) by four trained examiners (AMK, IU, VW, and RS). Only teeth with concordant ratings assessed by all four examiners were utilised. All carious lesions were visible in moist and dried states and could be classified as ICDAS code 2.^29^ Teeth with other ICDAS codes (0, 1, and 3–6) were excluded.

### Cleaning and storage

The roots were carefully purified from calculus, biofilm, and soft tissues using an ultrasonic scaler (Sinius; Sirona Dental Systems, Bensheim, Germany); occlusal surfaces (in particular, the carious areas) were excluded from sonicating to prevent lesion surface breakdown. After placing all teeth into an ultrasonic bath (Elmasonic S 130 H; Elma Schmidbauer, Singen, Germany) for 25 min, the crowns were cleaned by means of a rotating prophylactic brush (CleoProphy Brush, spiky; Zhermack, Marl am Dümmer, Germany) and prophylactic paste (Cleanic, fluoride-free; Kerr, Bioggio, Switzerland) for 10 s. Using this approach, the surfaces were cleaned without any damage. Subsequently, the teeth were carefully rinsed with an air/water sprayer (Sinius; Sirona Dental Systems, Bensheim, Germany) for approximately 20 s to eliminate all paste, powder, and (non-)natural deposit residues. Finally, all occlusal surfaces were thoroughly dried with compressed air for 30 s (Sinius; Sirona Dental Systems, Bensheim, Germany), and photographs of all teeth were taken (EOS 450 D, Macro Ring Lite MR-14 EX; equipped with a Macro Lens EF 100 mm, 1:2.8 USM; Canon, Tokyo, Japan). Until further examination, the teeth were stored in isotonic saline (0.9% sodium chloride solution; in-house production) using a storage box (Hornbach, Krems an der Donau, Austria) at room temperature.

### Baseline analysis

**Figure 1:**
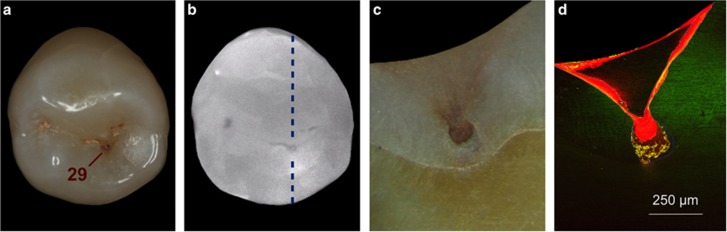
**Representative specimen of the RI/CR combination group**. (**a**) Macroscopic view of incipient occlusal lesion before treatment (note location of the highest DIAGNOdent pen value measured). (**b**) Radiation-free DIAGNOcam image of the same specimen, depicting the extent of the carious lesion, along with the sectional plane (indicated by the blue dotted line). (**c**) Macroscopic view of the same specimen, cut through the central grove, and depicting the fissure caries. (**d**) Corresponding CLSM micrograph (× 4), revealing the deep and homogeneous penetration of the resin infiltrant into the lesion body, visualized by the fluorescently labelled (RITC incorporated by polymerization) resin infiltrant, at the same time depicting the adhesive seal on the fissure walls. CLSM, confocal laser-scanning microscopy; CR, composite resin; RI, resin infiltrant; RITC, rhodamine B isothiocyanate.

Digital Imaging Fibre Optic Transillumination (DIAGNOcam; KaVo Dental, Vienna, Austria) was used as a radiation-free, handheld, fluorescent laser device for caries detection and ensured the full range of the extent of the occlusal lesions, and images were captured according to the manufacturer’s instructions. The teeth were fixed with the aid of an impression material (Plurasil Putty; Pluradent, Vienna, Austria) combined with a gingival mask (elastic replacement gingiva, AN-4 WUKV; Frasaco, Tettnang, Germany). Thus caries extent could be visualised (Figure 1a and 1b), and radiographs to detect the demineralisation were dispensable.^30^

Each cleaned and dried occlusal fissure system was measured by means of the cylindrical fissure probe of a laser-induced caries detection device (DIAGNOdent pen; KaVo Dental, Vienna, Austria) according to the manufacturer’s instructions. After calibration using a ceramic standard, the healthy surface of each tooth was set to adapt the machine onto the tooth. Then the pits and fissures were scanned with the DIAGNOdent pen until the areas with the highest peak values were identified (Figure 1a). All carious lesions with values “>17” were included.^31^

### Pretreatment

Prior to the treatment, the teeth (*n*=40) were deproteinised for 2 min using a bath of 2% sodium hypochlorite solution (NaOCl 2% Apotheke zum goldenen Engel, Graz, Austria) at room temperature.^32^ For this purpose, each tooth was placed in a cup (drinking cups, #900-8366; Henry Schein, Melville, NY, USA) and submerged in NaOCl (2 s). After this procedure, the teeth were rinsed off with an air/water sprayer (30 s) and dried with oil-free compressed air (30 s). Then all teeth were ready for infiltration and/or sealing.

### Allocation

**Figure 2:**
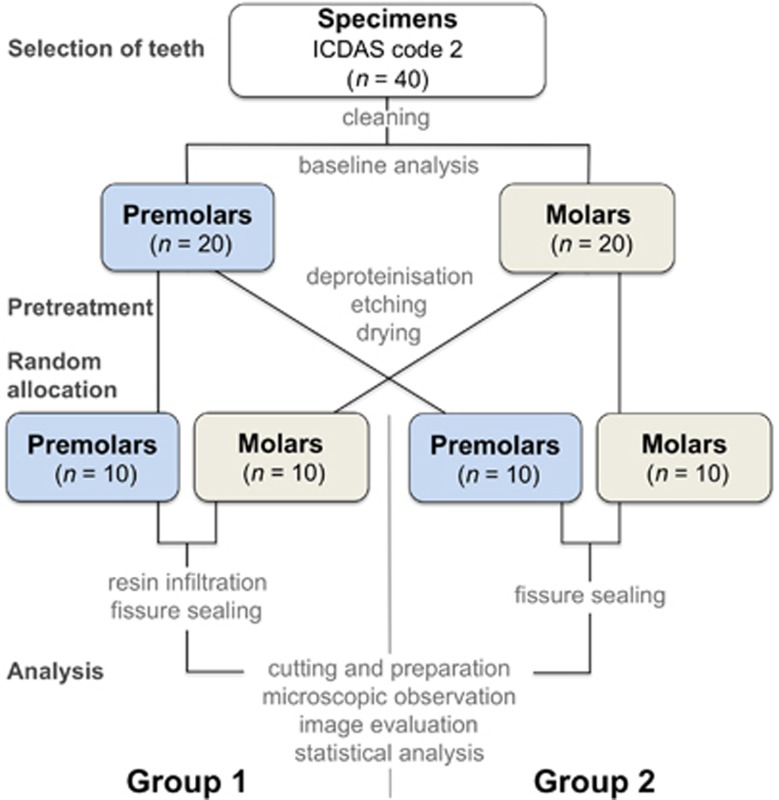
**Flowchart presenting group assignment and experimental set-up**.

Twenty premolars and 20 molars were randomly divided into two groups according to the following infiltration/sealing procedures (Figure 2). In Group 1 (10 premolars and 10 molars), both the RI (Icon; DMG, Hamburg, Germany) and the fissure sealant (G-ænial Flo A3.5; GC Europe, Leuven, Belgium) were applied. Group 2 was the control group (10 premolars and 10 molars), and only G-ænial Flo (GC Europe, Leuven, Belgium) was used to seal the fissures.

### Treatment

The RI (Icon; DMG, Hamburg, Germany) was used according to the manufacturer’s instructions. Initially, the pits and fissures of Group 1 were etched with 15% hydrochloric acid gel (HCl, Icon-Etch; DMG, Hamburg, Germany) for 2 min. Then the HCl gel was rinsed off with an air/water sprayer for 30 s, and the lesions were dried with oil-free compressed air for 30 s. All teeth were dried with Icon-Dry (99% ethanol; 30 s; DMG, Hamburg, Germany) and compressed air (30 s). Two drops of the infiltrating material were labelled with 10 μl of a red fluorescent dye (0.1 mmol rhodamine B isothiocyanate (RITC), excitation wavelength 570 nm/emission wavelength 595 nm; Babenberger Apotheke, Vienna, Austria).^33^ Then the prepared RI (Icon-Infiltrant; DMG, Hamburg, Germany) was applied with application brushes (Microbrush plus, superfine white; *Ø* 1.0 mm; Microbrush International, Grafton, WI, USA) to the etched and dried lesion for 3 min. Next, any surplus was wiped away with a soft foam pellet (*Ø* 4 mm; Henry Schein, Melville, NY, USA), and the resinous material was light-cured for 40 s. This curing was assured by placing the polymerisation unit (Silverlight Cordless LED Curing Light Unit, >1 100 mW·cm^−2^; Mectron Dental, Carasco, Italy) perpendicular to the lesion (and with a distance of 4 mm). The infiltration procedure was repeated with a penetration time of 1 min. Again, surplus was removed with a foam pellet (Henry Schein, Melville, NY, USA), and the RI (Icon-Infiltrant; DMG, Hamburg, Germany) was polymerised for 40 s. Finally, a fissure sealant (G-ænial Flo; GC Europe, Leuven, Belgium) was applied to each surface and light-cured for 40 s.

The control group received a sealant procedure only. First, the pits and fissures were etched with 15% hydrochloric acid gel (Icon-Etch; DMG, Hamburg, Germany) for 2 min, and the lesions were dried with Icon-Dry (DMG, Hamburg, Germany; 30 s) and oil-free compressed air for 30 s (compare protocol of Group 1). Subsequently, the pits and fissures were sealed using a non-labelled flowable (with a precuring penetration time of 30 s; G-ænial Flo; GC Europe, Leuven, Belgium; light-cured for 40 s). An extra layer of the infiltrant mixed with RITC was applied and polymerised to constitute the external border of the sealant for CLSM imaging (FLUOVIEW FV1000; equipped with a UPlanSApo, × 4 objective; Olympus, Tokyo, Japan). Areas of special interest were analysed at higher magnifications (× 20 objective).

### Cutting of specimens

All teeth were completely sectioned in the bucco-lingual direction through the occlusal lesion. For this purpose, every tooth was flattened with abrasive paper (800 grit; sia Abrasives Industries, Frauenfeld, Switzerland) on the mesial or distal side. Then, it was possible to use superglue (Fix All Crystal; Soudal, Turnhout, Belgium) to attach teeth to specimen holders (Objektträger; Bresser, Rhede, Germany). By using the peak value from the DIAGNOdent pen (fissure probe; KaVo Dental, Vienna, Austria), it was possible to determine the appropriate cutting position. Each tooth was horizontally mounted and severed under copious water-cooling. A cut-off wheel (942.104.200; Brasseler, Lemgo, Germany) was fixed using a straight handpiece (T1 Line, H40 L 1:1; Sirona Dental Systems, Wals bei Salzburg, Austria) that was mounted on a modified grinding table (Bohr- & Fräsbank BFB 2000; and Kreuztisch KT 150; Proxxon, Föhren, Germany). Using an acrylic resin (Futura Gen Acrylic; Schütz Dental, Rosbach, Germany), the slides with the fixed teeth were attached to the modified grinding table, thus allowing for precise crosssections.

After each tooth was severed, abrasive papers (800, 1 200, 2 500 grit; sia Abrasives Industries; 5 000 grit; Starcke, Melle, Germany) were used to approach the most central point of the lesion (Figure 1c), and this process was constantly controlled by means of magnifying glasses (TTL Lupe 2.0 × 350 mm; American Dental Systems, Vaterstetten, Germany).

All sectioned lesions were cleaned using an ultrasonic bath (Elmasonic S 130 H; Elma Schmidbauer, Singen, Germany) for 10 min. In both groups, the non-infiltrated areas of the lesions were stained using a green coloured fluorescence dye (0.1 mmol fluorescein isothiocyanate (FITC), excitation/emission peak at 490/520 nm; Babenberger Apotheke, Vienna, Austria). After imbibition for 2 h, FITC surplus was removed with foam pellets (Henry Schein, Melville, NY, USA).

### Microscopic observation and image evaluation

All specimens were studied using CLSM (FLUOVIEW FV1000; Olympus, Tokyo, Japan), and the captured CLSM images were evaluated by means of an open-source image editor (GIMP 2.8.18, GNU Image Manipulation Program; http://gimp.org).^34^ Within the non-infiltrated lesion area (and within the sound enamel), 10 measurement points each were randomly selected to determine the auto-fluorescence of the specimens (both red and green channels). Thus it was possible to clearly define the infiltrated areas and the lesion outlines, and these measurements were not distorted by the background noise from the specimens’ auto-fluorescence. The caries lesion was bordered using GIMP’s dedicated “free selection” tool. With the help of the measuring tool (“histogram”), the number of pixels within the marked range was determined.

### Measured variables and statistical analysis

Total enamel lesion sizes (LS_Enamel_) and infiltrated lesion areas (ILA_Enamel_) were calculated, and the percentage of proportions of infiltrated lesion areas (% ILA_Enamel_) were computed (% ILA_Enamel_=ILA_Enamel_ × 100 ÷ LS_Enamel_). Raw data were entered into Excel sheets (Microsoft, Redmond, WA, USA), and all analyses were performed by means of a statistical software package (SPSS 23.0; IBM, Armonk, NY, USA). Data were analysed with respect to the normality of the distribution using Kolmogorov–Smirnov test. Mann–Whitney test was used to compute intergroup comparisons, and paired intragroup samples were analysed using Wilcoxon’s test. A *P*-value of <0.05 was considered statistically significant.

## RESULTS

From the total of 40 macroscopically non-cavitated occlusal lesions (20 premolars and 20 molars) used for the present study, one specimen (premolar, Group 1) was lost owing to preparation damage. With one exception (percentage values of resin infiltration; *P*=0.200; Kolmogorov–Smirnov), the data were not normally distributed. Mean pixel values ±standard deviation (SD) of LS_Enamel_ were 241 638.5 px ±257 482.2 px in Group 1 and 88 749.0 px ±93 373.3 px in Group 2, and these differences were significant (*P*=0.012; Mann–Whitney). Mean values ±SD of LS_Enamel_ from all specimens were 90 055.2 px ±74 924.1 px for premolars (Groups 1 and 2) and 232 753.1 px ±260 294.1 px for molars (Groups 1 and 2). There was no significant difference between premolars and molars in both groups (*P*=0.092; Mann–Whitney). In Group 1, the extensions of the premolar lesions were significantly smaller compared with the dimensions of the molar lesions (*P*=0.004; Mann–Whitney).

Significant differences between Groups 1 and 2 were revealed with regard to the infiltrated areas. Within the control group (Group 2), no parts of the carious lesions were infiltrated by the fissure sealant (*P*<0.0001; Wilcoxon). Regarding Group 1, the mean ±SD of the infiltrated areas was 81 684.1px ±89 020.8 px, and which corresponds to 45.9% ±24.8% of the entire lesion extension (% ILA_Enamel_). These results indicate that resin infiltration filled major parts but did not completely occlude the carious lesions (see Figure 1d). Significant differences between the infiltrated areas and complete lesion areas were observed in premolars (57.9% ±23.1% *P*=0.008; Wilcoxon) and molars (35.3% ±22.1% *P*=0.005; Wilcoxon).

**Figure 3:**
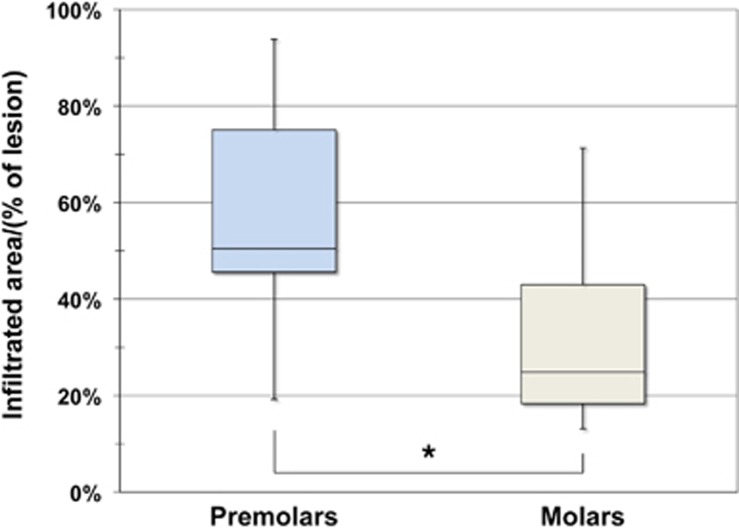
**Box-and-whisker plots (100%) presenting infiltrated areas (in % of total enamel lesion extent) of Group 1**. Boxes represent the interquartile range (IQR); the lines inside represent the median, while whiskers denote the maximum and minimum values. Subgroups correspond to bicuspids (*n* = 9) and molars (*n* = 10), and percentage of infiltrated areas differed significantly (*P = 0.034) between both tooth types. (Due to the missing infiltration capacity of the flowable composite resin, results of Group 2 are not depicted).

With respect to the pixel numbers, no significant differences regarding the total infiltration between premolars (52 553.6 px ±40 757.8 px) and molars (107 901.6 px ±112 962.3 px) were observable in Group 1 (*P*=0.102; Mann–Whitney). By contrast, mean percentage values (% ILA_Enamel_) differed significantly (*P*=0.034; Mann–Whitney). The corresponding box-and-whisker plots are provided in Figure 3.

**Figure 4:**
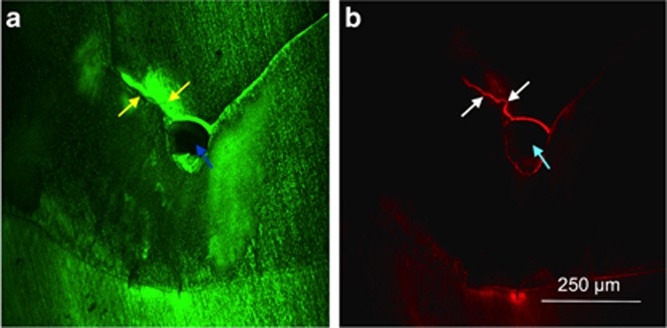
**CLSM micrographs (× 4) depicting a specimen from the CR group**. (**a**) At the bottom of the fissure, an unfilled area (void) is clearly visible (see blue arrows), along with the fluorescently labelled (FITC) porous volume of the carious lesion. (**b**) Microleakage visualized by fluorescent labelling (RITC, see yellow arrows). CLSM, confocal laser-scanning microscopy; CR, composite resin; FITC, fluorescein isothiocyanate; RITC, rhodamine B isothiocyanate.

**Figure 5:**
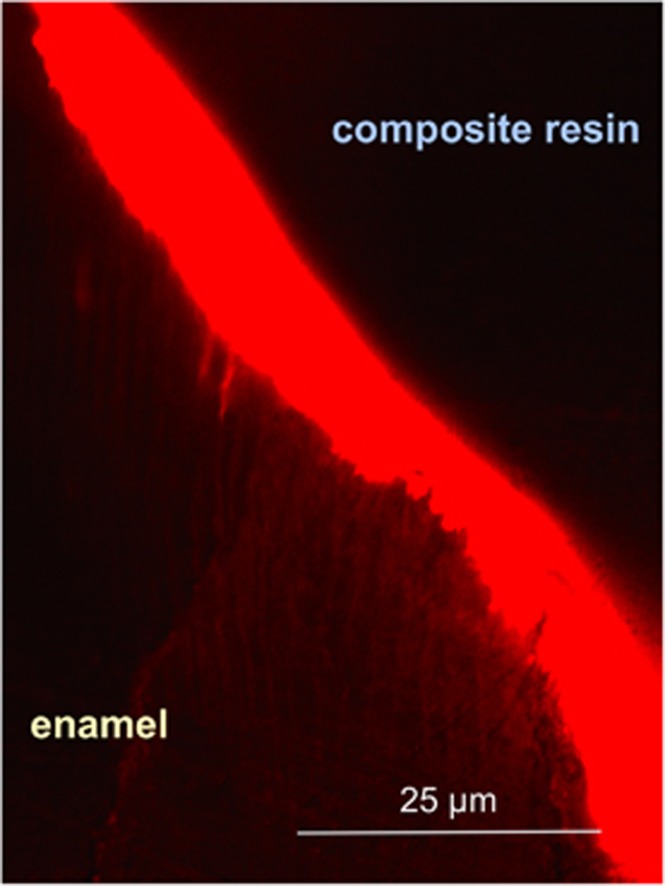
**CLSM micrograph (× 20) of infiltrant/enamel interface (aspect corresponding to the specimen known from Figure 1d)**. Typical for the RI/CR combination group, penetration into etched/eroded enamel, (interprismatic) tag formation and mechanical interlocking are clearly visible and indicate the integrity of the bonded interface.

Microleakage was observed more often in the CR group, and a typical example is provided in Figure 4. Conversely, the RI/CR combination resulted in a scattered specimen exhibiting an interfacial gap (Table 1) and notable marginal adaption (Figure 5). No entrapment of air was detected within the sealant material. In Group 2, voids were observed at the bottom of the fissures in 17 specimens. By contrast, voids on the fissure ground were noted in only two specimens in Group 1 (Table 1).

## DISCUSSION

The objective of the present study was to evaluate the efficacy of combining resin infiltration and conventional fissure sealing (using a thixotropic microfilled flowable CR) of occlusal carious lesions.Flowable CRs are widely used for fissure sealing^13, 35, 36, 37^ and exhibit increased retention rates and better physical properties compared with conventional fissure sealants.^37^ In the present study, the CR was used according to clinically established practice but completely failed to penetrate into the initial occlusal lesions. By contrast, the application of the RI before performing the fissure sealing revealed a constant and high degree of infiltration of all carious lesions (ICDAS code 2). Thus our null hypothesis was rejected.

Fluorescence-based systems are effective for caries diagnosis. In the present study, however, visual screening according to ICDAS was the main criterion to select the respective teeth, and this screening was performed by mutual agreement of the four participating examiners. Visual inspection is more reliable and well reproducible compared with laser fluorescent techniques;^38^ therefore, DIAGNOdent pen^31^ and DIAGNOcam^30^ were used as secondary diagnostic adjuncts and to locate the respective area for severing the teeth in preparation of the CLSM part of this investigation.

As recently demonstrated, inactive occlusal carious lesions remain stable over a 1-year period and do not need more attention than sound occlusal surfaces;^39^ this finding clearly confirms the maxim of Elderton (stating in 1985 that “if being in doubt, prevent and see”).^40^ However, regarding a treatment decision, sealing of pits and fissures is efficacious in reducing the progression of non-cavitated carious lesions in permanent teeth of children, adolescents, and young adults^2^ and exhibited favourable results even with cavitated carious lesions.^5^ Notwithstanding, microinvasively sealed therapies are at risk for measurable failure rates of up to 10% per year^8^ and are considered prone to higher retreatment needs compared with minimally invasive therapies.^41^ This feature calls for the utmost vigilance. Although occasionally considered a simple procedure, fissure sealing clearly requires cognitive capabilities (including caries risk assessment at both the patient and the tooth/surface levels). Moreover, in-depth knowledge about anatomical and biological issues and material science are deemed necessary. Finally, operative skills and due care are essential given that placing of sealant is technique sensitive.^42^

In particular, patients at high risk for caries should benefit from any therapeutic regimen; however, considerable differences between practitioners’ treatment decisions have been reported with regard to occlusal carious lesions.^43^ From a scientific perspective, it is well accepted that any surgical restorative intervention should be regarded as a last resort only,^29^ and timely recommendations for the type of lesions included in the current study unambiguously opt for a microinvasive sealing regimen.^8, 9, 29, 44^

In the present study, a meticulous surface cleaning (including deproteinisation using sodium hypochlorite^27, 32^) was performed in both groups. This procedure aimed at removing prophylaxis paste remnants^45^ and/or organic residues (for example, salivary components, acquired pellicle, biofilm, debris)^11, 46^ occluding the fissure entrance and provided improved access for the following treatment steps. Via liquefaction of organic material (both on and within the enamel lesion), sodium hypochlorite (if used *ex ante*) will approximately double the retentive surface of etched enamel.^47^ Thus both the quality of the etching pattern and the sealant bond strength will be improved,^47^ simultaneously significantly reducing the rate of microleakage of the succeeding sealant application^42^ and increasing infiltration depths.^27^

The use of hydrochloric acid (instead of other acids) has been recommended for pretreatment of the pseudointact surface layer of incipient lesions to be infiltrated^20, 48^ and is advantageous with inactive lesions.^49^ Where applicable, HCl may be combined with abrasives.^19^ However, it seems worth emphasising that a complete removal of the surface layer is not considered the primary objective of the infiltration concept; instead, increasing the penetrability by opening the porous system of incipient lesions is essential. The surface layers of active carious lesions exhibit a thickness of approximately 50 μm.^50^ With sound or demineralised enamel, up to 30 μm erosion will result after HCl-etching, along with typical etching patterns.^51, 52^ Moreover, even aprismatic enamel layers characterising the occlusal fissure systems can be etched and removed partially, thus rendering enamel prisms identical to those of normal enamel.^53^ The prismless enamel layer of occlusal fissures is acid-resistant (thus delaying the onset of caries),^54^ and etching efficacy has been attributed to the strength of the used acid (and to the etching time).^55^ From a series of experiments, it became clear that a regular etch pattern with increased surface roughness is possible at the very base of pits and fissures,^54^ thus providing the basis for adhesive linkage. Therefore, mechanical removal of aprismatic enamel (by enameloplasty or fissurotomy), though favourable in some cases,^56^ would seem dispensable,^35^ whereas etching (using etch-and-rinse systems) is regarded as essential for adequate mechanical adhesion and minimising loss of retention.^55, 56^

Acid etching of fissures leads to a pronounced reduction of bacterial counts^3^ and increases both roughness and free energy of the surface,^12^ thus enhancing the reactivity of enamel. Upon conclusion of the drying procedure, all specimens included in the present study were prepared. Accurate desiccation (using drying agents such as alcohol or acetone) is considered essential for infiltration but should be included in any sealing procedure because penetration will be essentially enhanced.^57^ Although pretreatment conditions (cleaning, deproteinisation, etching, and drying) were identical for both groups, the capillary forces enabled the infiltration of the low-viscous resin in Group 1 due to the high coefficient of penetration (being inversely proportional to its viscosity).^1, 58^ Thus a pronounced interlocking between RI and enamel surface could be established (Figure 5), and this finding was consistent with a recent study reporting on deep infiltrant penetration into etched enamel.^59^

It has been argued that because of their lower viscosity and better flow characteristics, unfilled resins penetrate deeper into the fissure system, thus achieving increased retention rates compared with (partially) filled sealants or flowables.^1^ As far as narrow and deep fissures are concerned, total sealing will depend on the sealant’s flow capacity.^11, 13^ Indeed, low-viscous sealants exhibited better marginal adaptation compared with materials of high viscosity in some studies.^12, 60^ Consistent with a recent examination,^20^ the current investigation (using the thixotropic G-ænial Flo) confirmed the poor outcome of highly viscous materials.^61^ Voids, porosities, and microleakage were observed mainly in the CR Group (Figure 4 and Table 1), and this finding might jeopardise such treatment regimens in the long term. However, it should be emphasised that the ability to penetrate alone obviously does not affect the respective retention rates.^62^ Instead, other factors (distinctive anatomic features^13^ or moisture control^62^) have a negative influence in the long term.

Some clinical trials have indeed elaborated significant failure rates of sealed fissures of approximately 30% (depending on the observation time),^36, 63^ thus indicating high needs for repair.^41, 61^ Regarding the outcome of timely sealant materials, several shortcomings have been identified, and these limitations include pretreatment modalities of enamel,^12, 55, 57, 60^ curing modes,^60^ and viscoelastic properties of the material.^12, 13, 36, 37, 60^ Polymerisation contraction and hygroscopic and thermal expansion of fissure sealants^64^ might lead to microleakage and gap formation, whereas filler content should affect fracture resistance.^1^ Finally, wear and abrasion, surface degradation, and deterioration of the matrix-filler interface contribute to (partial) loss of retention or sealant damage over time, subsequently allowing for re-ingress and in-depth migration of bacteria and consequently constituting a site of potential caries-susceptibility.^1, 10, 37^ Thus, given that gap-free interfaces are not always guaranteed, vigilant follow-up assessments (including repeated individual instructions concerning continued biofilm removal) and ongoing and proper monitoring of the sealed surfaces is imperative albeit challenging.

To improve the quality of fissure sealing (usually using more or less viscous materials), the use of an intermediate bonding layer between sound enamel and sealant has been advocated.^44, 61^ Thus bond strengths of sealants will be increased, and microleakage is reduced. Furthermore, this concept facilitates flow of any viscous sealant material, reduces the emergence of possible voids, and improves retention of the complete restoration. These features were confirmed by the present results. Only two specimens of the RI/CR combination group exhibited any voids, thus indicating that the RI approach had transformed all fissures into shallow fissures (being easily sealed thereafter; compare Figure 1d). Without a doubt, complete sealant retention will control caries onset given that microbial metabolism (and acid production) will be discontinued by the suspended availability of nutrients.^10^ Although sealing of pits and fissures can be regarded as part of a comprehensive caries-preventive approach, it should be emphasised that leakage or incomplete retention of a sealant does not inevitably result in carious lesions. Thus, retention rates *per se* are a meaningless outcome measure.^65^

The thoughts mentioned above do apply in particular to sound teeth. However, current evidence suggests that sealants are also effective with regard to secondary prevention; therefore, fissure sealing has been advocated for early non-cavitated carious lesions.^29^ Regarding this aspect, the present study clearly demonstrated material-based improvements given that the low-viscous infiltrant used penetrated deeply into the respective carious lesions (Figure 1), thus occluding its porous volume. The use of the infiltrant as an intermediate coupling agent compatible with conventional adhesives and CRs as a beneficial pretreatment in demineralised enamel^66^ (simultaneously penetrating enamel eroded by acids^59^) strengthened the entire restoration by establishing appropriate adaption and adequate adhesion into the etched fissures. The latter has recently been identified as a key consideration for successful sealing.^37, 44^

Unfilled resins exhibit high polymerisation shrinkage. With this feature in mind, it should be noted that the fissure morphologies used in the present study are generally comparable to Class I cavities and are subsequently associated with polymerisation stress on the bonded fissure surfaces due to the high *C*-factor of such cavity geometries. The latter might lead to detachment and leakage;^57^ therefore, we attempted to address these shortcomings with the repeated application of the infiltrant (3 min plus 1 min). As noted in Figure 5, adhesion to the etched enamel was successful. In total, only one out of 19 specimens in Group 1 exhibited some form of microleakage (see Table 1). Therefore, a three-dimensional analysis would have provided more detailed insights,^11^ but this process was not possible with the present set-up.

The current outcome clearly revealed that penetration into eroded enamel and infiltration of carious lesions using a low-viscous infiltrant can be successfully merged with conventional flowable resins, thus conforming to the requirements previously defined.^12^ Moreover, owing to the oxygen-inhibition layer of the light-cured infiltrant,^67^ the flowable CR could cross the interface and form an interdiffusion zone (Figure 5), thus presenting a top coat^32, 52^ and compensating for the infiltrant’s surface properties.^67, 68^ This combination provided well-adapted and adhesive anchoring that rendered the fissure system impervious to further demineralising attacks,^69^ as recently demonstrated with clinically infiltrated and sealed primary molars.^21^ Although the initial sealing quality was improved by the present approach, whether the increased penetration depths of the used RI/CR combination will result in sufficient sealant retention over time would be interesting. Therefore, further studies are clearly warranted.

The infiltrant used in the present study must not be confused with conventional or self-etch adhesive systems. The latter typically reveal reduced retention time compared with conventional sealing.^55^ Instead, it should be borne in mind that the primary beneficial effect of the RI is due to its inherent penetration capabilities, thus occluding the porous volume of carious lesions and achieving superior results compared with other adhesive bonding agents.^58, 68^ Hence, although fissure sealing as a resinous top coat provides an impermeable diffusion barrier, the infiltrated lesion itself constitutes an obstacle located inside the lesion with a safeguarding function in case of breakdown or extensive wear and abrasion of the sealant.

Moreover, infiltration of initial lesions will significantly strengthen the hardness of demineralised areas,^17, 18^ thus providing a sound basis for any sealing restoration and potentially resulting in decreased failure rates. This feature is considered a further aspect of relevance. At this point, it should be re-emphasised that conventional caries removal only ensures a solid basement for any intended restorations.^8, 70^ With the present approach, carious lesions of premolars could be infiltrated by >50%, whereas molar lesions reached mean infiltration depths of approximately 35%, which was due to the advanced lesion sizes of the molars. Moreover, pits and fissures have been partially filled up with the RI (compare Figures 1 and 5). Accordingly, non-removed initial carious lesions that have been infiltrated and hardened would act as stress breakers,^44^ thus avoiding any repeated pressure forging of the polymerised sealant (a phenomenon known as “trampoline effect”), exhibiting better fracture resistance compared with traditional restorative methods,^71^ and leading to prolonged longevity of the final restoration.^70^

Although the infiltration of proximal caries has been established as a stand-alone solution,^15, 16^ the same is not recommended for occlusal lesions. The commercially available product is a low-viscosity adhesive resin intended to penetrate the porous volume of initial caries, thereby resulting in a caries-resistant unit composed of a mixture of infiltrant and enamel prism remnants.^72^ After intended use, infiltrant surplus has been described as very thin resin coats^72^ and would seem suspicious to increased oxygen-induced polymerisation inhibition and poor monomer conversion,^73^ which is prone to rapid abrasive loss due to mastication and brushing. By contrast, massive surplus (fill-up of fissures) should result in polymerisation shrinkage of the non-filled infiltrant. To overcome this drawback, the addition of filler components to the infiltrant has been suggested recently^74^; however, further aspects of this approach (for example, mechanical strength, wear behaviour, adhesive characteristics) that remain unknown to date require resolution before any definite recommendation can be provided. In total, the shortcomings noted above can be easily avoided using a supplemental layer of a flowable composite, thus sealing the fissure system, acting as an additional diffusion barrier against nutrients and acids, and serving as a top coat enhancing the infiltrant’s surface qualities.^32, 52^

When reflecting on the infiltrated lesions, the improved adaption, and the enhanced internal integrity of the restorations, the results of the current study would favour the RI/CR combination for sealing deproteinised non-cavitated occlusal lesions. By adopting this therapeutic strategy, the recently reported retreatment needs of microinvasive sealing^41, 61^ should be reduced, thus warranting both the increased costs and the complexity of this combined treatment approach. Undoubtedly, the encouraging outcome of the present study justifies additional future experimental and clinical studies and long-term investigations focusing on permanent dentition.

## CONCLUSION

Considering the limitations of the present study, the following conclusions can be drawn:

- The use of a RI is an effective method to occlude the porous volume of deproteinised and etched occlusal subsurface lesions (ICDAS code 2).
- Resin infiltration of occlusal caries provides a stable basis for flowable CRs used for fissure sealing.
- The RI can deeply penetrate into HCl-eroded enamel of occlusal fissures. In combination with a flowable CR, the infiltrant material has a positive effect on marginal adaption and internal integrity of fissure sealing of carious (pre)molar fissures, thus ensuring increased protection against microleakage and loss of retention.

Therefore, the RI/CR combination clearly improves the initial quality of fissure sealing compared with the exclusive use of a conventional fissure sealant material and is recommended for clinical use, particularly with initial carious lesions.

## Figures and Tables

**Table 1:** Results regarding marginal adaption and (internal) integrity

	Group 1		Group 2	
	Premolars (*n*=9)	Molars (*n*=10)	Premolars (*n*=10)	Molars (*n*=10)
Microleakage	1	0	2	3
Voids within the fissure sealant	0	0	0	0
Voids at the bottom of the fissures	0	2	7	10
